# Design and piloting of a proposal for intervention with educational robotics for the development of lexical relationships in early childhood education

**DOI:** 10.1186/s40561-023-00226-0

**Published:** 2023-01-20

**Authors:** Verónica Moreno Campos, Francisco José Rodríguez Muñoz

**Affiliations:** 1 Departamento de Pedagogía, Didáctica de las Ciencias Sociales y Didáctica de las lenguas, Facultad de Ciencias Humanas y Sociales, Universitat Jaume I, Avda. de Vicente Sos Baynat s/n, 12071 Castelló de la Plana, Spain; 2 University of Almería, Didáctica de la Lengua y la Literatura, Carretera Sacramento, s/n, 04120 La Cañada de San Urbano, Almería, Spain

**Keywords:** Educational robotics, Lexical relationships, Communicative competence, L1 education, Twenty-first-century competencies

## Abstract

An applied research proposal for integrated learning based on the use of educational robotics has been proposed. The design has been implemented with a sample of 21 four-year-old students applying twenty-first-century competencies (collaboration, creativity, critical thinking, and communication) to learn the curricula related to the development of lexical relations. This research aims to apply data directly derived from the application of educational robotics in the classroom. The research aims focus on two fundamental questions: on the one hand, to verify whether the use of educational robotics in teaching practice is related to greater conceptual achievement and, on the other hand, to validate whether students apply transversal competencies through educational robotics. The results allow us to affirm that the didactic application of robotics activities achieves a high degree of conceptual integration when establishing lexical relationships and allows students to put into practice key transversal problem-solving and critical thinking competencies.

## Introduction

Twenty-first-century society can be defined as a culturally connected society due to technology. It is logical that in the field of education, more and more studies are interested in the integration of STEM disciplines (science, technology, engineering, and mathematics) as a way of acquiring what have been called twenty-first-century competencies: collaboration, creativity, critical thinking, and communication (Hussein et al., 2019). In a world where technology is present in all aspects of everyday life, teaching must be updated and implement new forms of competency learning where students are not limited to memorizing content but can learn the concepts thanks to the application of new technologies (Barrera Lombana, 2015; Anwar et al., 2019; Sánchez-Tendero et al., 2019; Turan & Aydogdu, 2020). In this sense, one of the most critical challenges facing the educational community is precisely knowing how to integrate educational robotics (hereafter, ER) into the design of classroom activities (Nikolopoulou & Gialamas, 2015; Papadakis, 2020).

ER is an emerging field in education characterized by the use of robots as learning tools capable of linking the different areas of the school curriculum with action competence learning. ER is conceived not only as an end in itself but as a way of enabling problem-based learning, where students must collaborate to solve challenges; in this way, their cognitive and communicative skills are enhanced around the meaningful learning of curricular content.

Most studies are framed within the constructivist paradigm (Piaget, 1968), according to which learning is constructed through processes of conceptual assimilation and accommodation through problem-solving. This paper accepts the constructivist statement as a starting point. However, it also integrates the precepts of the cognitive paradigm in which language is understood as another cognitive faculty that interacts with the other cognitive processes (i.e., memory or attention) by establishing a vehicular relationship. The communicative utterances of children when solving problems are important elements in determining how knowledge is acquired (Bruner, 1984). Another relevant notion is the fact that learning must be meaningful and transferable. In order for students to integrate knowledge into cognitive frameworks, we must allow them to experience and apply such learning to contexts that replicate or assimilate as much as possible to problems of everyday reality. Only in this way will the acquisition of so-called *learning and transfer skills* be achieved (Anwar et al., 2019).

However, one of the current limitations to integrating ER in the classroom is the lack of teacher training on the didactic possibilities of robotics (Canfarotta & Casado-Muñoz, 2019; Gökçearslan et al., 2018; Uğur-Erdoğmuş, 2021). Without a sound theoretical background and a foundation on which to base the didactic implementation of ER, the cognitive and pedagogical benefits derived from the application of robotics as an educational tool (Angeli & Valanides, 2020; García-Valcárcel & Caballero-González, 2019; Sullivan & Bers, 2016) will not be transferred to students. It is, therefore, urgent that ER research offers models of applied learning to the school curriculum that teachers can understand and apply (Acosta, 2016). It is, therefore, necessary that the type of learning be determined based on both the type of learner (infant, primary, secondary, high school, university) and the robot used (Jung & Won, 2018).

One of the skills pointed out by studies in robotics is the ability to divide the challenge into smaller tasks, the so-called sequential thinking, which, in the field of language learning, finds its methodological correlate in the *task-based approach* (Long & Crookes, 1992). As stated Langacker (1987), the notion of *profiling* plays a fundamental role in this skill wherein the application in ER would explain the fact that, in order to solve the challenge posed, students must be able to select from among all the stimuli received, those that are informatively relevant. Thus, knowledge is acquired by inferring its procedural utility by bringing various interdisciplinary skills into play. This type of integrated learning must be developed from the early stages of education when the child establishes his or her cognitive process. One learning approach that has been successfully implemented with kindergarten students is *Robotics Project-based Learning* (Papadakis, 2020). This proposal includes the approach of knowledge from interdisciplinary areas in its didactic method. With regard to the Spanish curriculum, Sánchez-Tendero et al., (2019) evaluated the motivation and enjoyment of students in the third year of kindergarten, and their degree of assimilation of a learning process included in the curriculum of the “Knowledge and Interaction with the Environment” area, using the Bee-Bot and Blue-Bot robots. The results indicate that using robotics as a means of learning is both useful and motivating. The benefits of ER in teaching processes have also been found in inclusive education. Likewise, the study by Hamzad et al. (2014) focused on the teaching process of preschool children with autism. They concluded that, through the use of ER, students managed to better generalize learning by facing challenges appropriate to their level that reproduced contexts closer to their reality than when learning was solely presented by visual or auditory means. Furthermore, the systematic review supported by Tlili et al. (2020) analyzed the design, implementation, and outcome of robot-assisted in special education research through the perspective of *activity theory*. This research underlines the importance of designing didactic activities by selecting objectives and robots appropriate to the possibilities and needs of the students.

Our proposal is framed within the inclusive school paradigm advocated by Daniela and Lytras (2019), where ER is conceived “as a tool for knowledge construction and as an assistive tool for students who have problems in specific fields, or ER may be used to change students' attitudes to learning-class culture-allowing everyone to be accepted and involved” (p. 222). We defend that ER is a motivating learning medium for students, who activate all their competence and communicative strategies to overcome the challenge posed by the teacher. In our case, we present an activity design in which students need to apply their knowledge of the semantic relations corresponding to the second year of Infant Education of the Spanish school curriculum called “Languages: Communication and Representation.” This area includes the linguistic items corresponding to the development and acquisition of 4-year-old children. Our proposal focuses on the semantic component, specifically, the categorical relationships established between concepts. When learning the concepts within our daily environment, inclusive categories are established that organize knowledge according to their similarities, thus obtaining semantic fields. By organizing meanings into *semantic fields* or *hyperonyms* (“a lexical unit, an umbrella term, that includes within it, the meaning of other words”), cognitive processing is optimized, since the categories are grouped around a common semantic feature (“a minimal semantic feature, a distinctive component of meaning that differentiates one lexical unit from another”). This cognitive saving, called *semantic priming*, has been investigated in the area of linguistic development, where several neuroimaging studies on cognitive processing have shown that, when a *hyperonym* appears in a text, it directly preactivates the related concepts: the *hyponyms* (Kandahai & Federmeier, 2008; Takashima et al., 2019; Mathur et al., 2020; Luchkina & Waxman, 2021). This categorical relationship between concepts is developed and established in the oral language of children between three and four years of age (Mueller & Cramer, 2001; Tomasello, 2003), wherein the child interacts with his environment and learns the relationship between the representational elements of the world. Thus, in the case of the hyperonym “animal,” the child groups the concepts related to the semas “+alive,” “−human,” “−vegetable” and links the concepts of “cat, lion, elephant…,” which in this case would be the co-hyponyms (“hyponyms that refer to the same hypernym or superordinate term”).

The approach of this study is innovative in that it presents an applied proposal that uses ER as a means of linguistic curricular learning integrated into teaching practice and where not only are learning outcomes measured, but also the viability of the didactic design. In the context of Spain, we found few proposals applied in Early Childhood Education courses focused on the area of “Communication and Representation of Reality.” The proposal by Hidalgo and Pérez-Marín (2019), whose objective is based on learning to exchange turns of speech in students aged 3–5 years, stands out. However, the methodology and results are not aimed at verifying whether learning had been achieved but rather verify whether the students enjoyed participating and were engaged with the didactic experience.

This research aims to apply data directly derived from the application of educational robotics in the classroom. Our research focuses on the shortcomings identified by Toh et al. (2016), Jung & Won (2018), and Hussein (2019) regarding ER research. It provides actual data to answer the following questions: (1) what knowledge has implemented educational intervention mediated by the use of ER, (2) what didactic objectives related to the curriculum can be utilized in a crosscutting manner thanks to the use of ER, and (3) to which characteristics do the young children's learning processes respond?

## Design of the educational intervention proposal

### Curricular framework

Our proposal is framed within the regulations governing the curriculum of Early Childhood Education in Spain (*Real Decreto 95/2022, de 1 de febrero, por el que se establece la ordenación y las enseñanzas mínimas de la Educación Infantil [Royal Decree 95/2022, of February 1, which establishes the organization and minimum teaching of Early Childhood Education]*). The contents are included in Area 3, “Communication and Representation of Reality,” and contribute to the development of communicative competence by focusing on teaching and learning the lexical relationships between the meanings of words.

### Activities

**Fig. 1:**
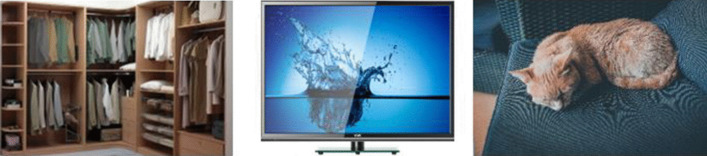
Association activity for the hyperonym “furniture”—hyponym “cabinet”

**Fig. 2:**
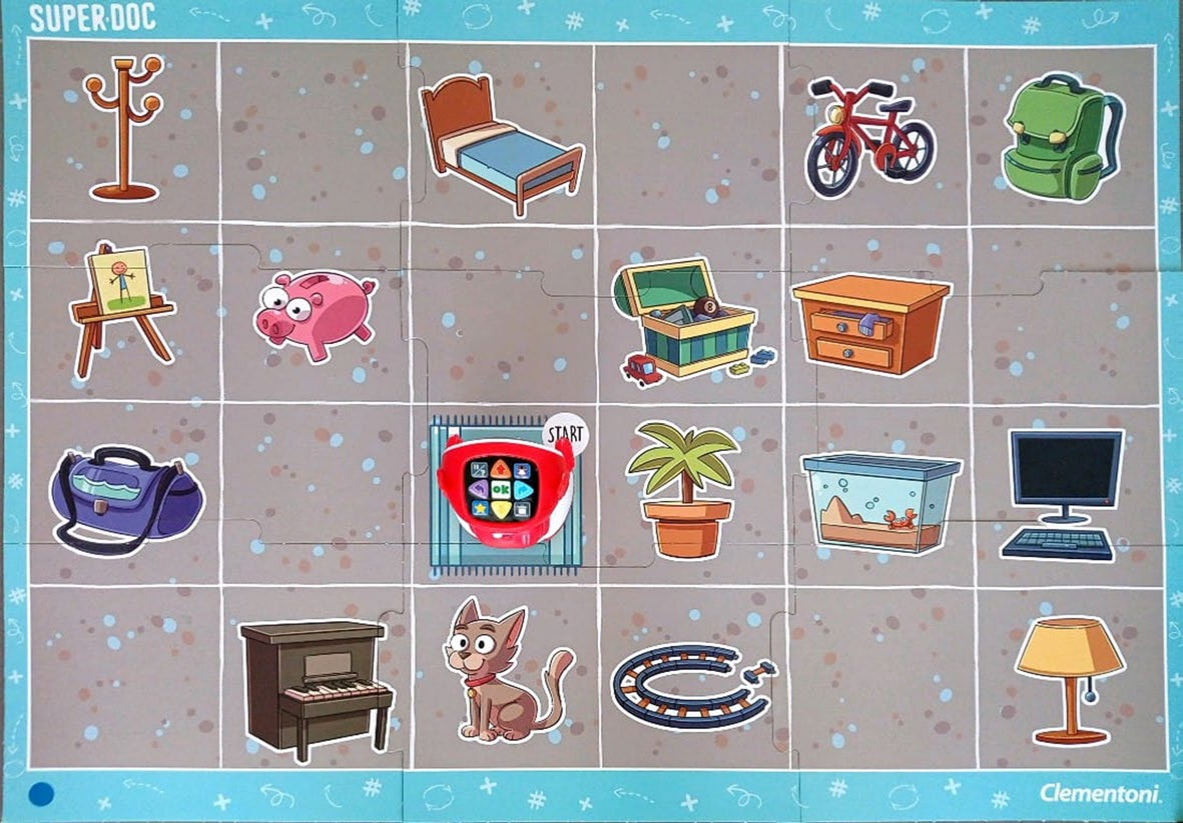
Clementoni’s board used for the ER activity

Ten activities were planned: five for the initial assessment and five for the final assessment. All activities follow the same scheme: Each child is individually presented with a hyperonym and must select the related hyperonym from three images. Only one of the three images is the correct answer (the one that shares the sema with the hyperonym). Of the remaining two responses, one shows some related seme, while the other does not share characteristics or semes. Figure 1 presents an example where the child must relate the hyperonym “furniture” with its hyponym “closet.” As distractors, the image of “television” (which shares the seme “homey”) and the semantically unrelated image “cat” have been included:

The educational robotics activity (ER) was carried out with a group. Each group then listened to a story narrated by the examiner and then help the protagonist of the story (the robot) to match a hyperonym, such as “means of transportation,” with its hyponym, “bicycle,” from the images represented on the board (Fig. 2):

## Materials and methods

### Participants

The study involved 21 children enrolled in a public nursery school in the city of Valencia (Spain): specifically, 11 girls and 10 boys between the ages of 4 and 5 *(M* = 4.5). All participants lived with their families and had a middle socioeconomic and cultural level. The students' legal guardians signed an informed consent form authorizing their children to participate in the activity.

### Instruments

**Fig. 3:**
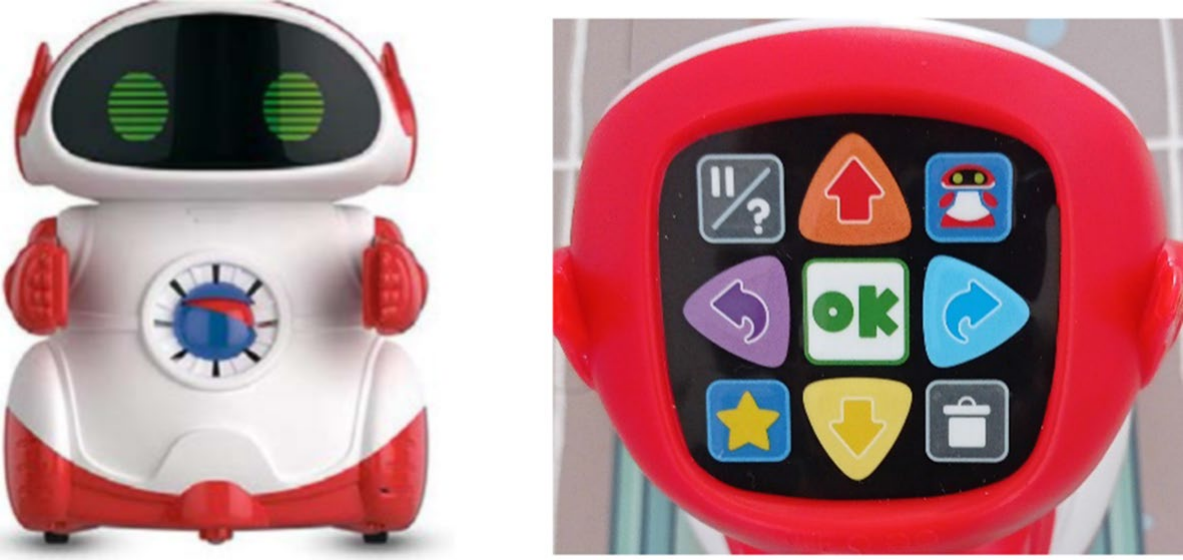
Robot and control panel

The initial and final knowledge assessment was carried out by selecting 15 × 25 cm images on a white background and without graphic aids. For the development of the ER activity, we used the material provided was by Super.Doc from the Clementoni publishing house: It contains a cardboard puzzle board with printed images and a robot without parts measuring 41.8 × 9.3 × 27.8 cm and 1.84 kg whose programming only allows spatial movements in a straight line of 15 cm and with the possibility of programming 45-degree turns (Fig. 3).

The TEPI scale (*Toy Effects on Play Instrument*, Trawick-Smith et al., 2011) was applied to qualitatively assess the degree of individual achievement of the objectives related to the construction of learning and the so-called twenty-first-century competencies: thinking and learning (knowledge construction, problem-solving) creativity, imagination, social collaboration, and independent use.

### Procedure

We followed the guidelines endorsed by Tejada (2005) and Mayor (2008) for the assessment of educational intervention programs according to the relationship between the purpose of the evaluation and the moment of learning, namely: initial assessment of prior knowledge, assessment of the development of the intervention proposal (Hervás et al., 2018), and final assessment of learning outcomes (Causo et al., 2015).

In the initial evaluation, the student's previous knowledge of the lexical relationship of words was assessed. The purpose of this evaluation was to lay the foundation for measuring the effectiveness of the intervention after analyzing the learning outcomes. To this end, five hyperonyms were selected, and each student was asked to select the related hyponym from a series of three images. Each series was composed of a semantically unrelated word, the correct answer, and a related word, either by analogy or in opposition (e. g., hyperonym “vehicle,” options: “stone,” “car,” “horse”). The number of successes was quantified, but also the type of failures. The teacher conducted the initial assessment in a 40 min session, and data collection was done on an Excel spreadsheet.

A multilevel analysis was carried out to assess the development of the intervention proposal. The methodological possibilities of the activity were initially analyzed, followed by an analysis of the didactic applications of the proposal (Hervás et al., 2018).

1. Methodological possibilities: applicability of the design, possibility of teamwork, and problem-solving capacity.
2. Didactic possibilities: ability to communicate among group members, cooperative work, and ability to design and plan the solution to the challenge posed.

The proposed intervention was carried out in two 40 min sessions. In the first session, three groups were assessed, and, in the second, four groups consisting of three randomly distributed students, but with the condition that they were constituted under the premise of sexual heterogeneity. Each evaluation was conducted in a room from the rest of the classmates to avoid interference. The researcher narrated a motor story where they had to help the robot solve five challenges to find the compass that would help him return to his planet. The five challenges were based on creating associations between a hyponym and its hyperonym, or vice versa. For example, the researcher posed: “the robot must catch its animal,” and the children had to associate the hyperonym with the image of the hyponym that appeared on the board: in this instance, “cat.” Once the semic relationship was found, the group had to arrange the movement arrows to organize the robot's movement, program it, and solve the next challenge.

In the final assessment, included in Table 1, the group successes were quantified. The nature of the failures was qualitatively assessed according to whether they were due to difficulties in establishing the seme relationship (related/unrelated) or to difficulties in programming the robot.

**Table 1:** Assessment of the intervention proposal

Purpose	Initial assessment	Developmental assessment	Final assessment
	Diagnostic	Formative	Summative
Target	Establishing the knowledge base of the students	Identify the key points of the training process Improve student learning	Certification of student progress Assess the adequacy of the educational intervention program Adapt the development of the program to the needs detected
Form of assessment	Number of successes Types of failures	Evaluation of methodological and didactic possibilities: TEPI evaluation scale	Count of successes Types of failures

## Results

The initial assessment aims to establish the students' knowledge base and analyze the difficulties in learning and consolidating inclusive lexical relationships. After the initial test, the average number of successes was 2.8. The highest percentage of errors pertained to choosing unrelated words (63%), while 27% identified incorrect concepts but with a semantic linkage.

When assessing the ER activity, the methodological and didactic possibilities of the approach were assessed through the TEPI scale (Trawick-Smith et al., 2011). Researchers qualitatively assessed the degree of performance of each student on a 5-point Likert scale based on twenty-first-century competencies:

Thinking and Learning:

- Constructing Knowledge: how students manifest the acquisition and assimilation of new knowledge through action in the proposed activity.
- Problem-solving: students show a variety of resources when solving the challenges posed.
- Inquiry: expressing interest and asking questions to satisfy the needs of the activity.
- Engagement: students show interest throughout the activity.

Creativity and Imagination:

- Creative Expression: use and diversity of oral expression (verbal and nonverbal).
- Imagination: how each child manifests creative thinking.

Social Interaction and Independent Use:

- Collaboration: how children manifest intergroup collaborative behaviors.
- Independent Use: how children demonstrate independent skills without peer or adult assistance.

Appendix 1 shows the TEPI scale results of each student's performance during the robotic activity. The examiners rated from 1 to 5 the degree of competency integration shown by each student. In the Thinking and Learning section, the averages obtained in Inquiry (3.95), Constructing Knowledge (3.76), and Engagement (3.67) stand out because they allow us to infer the degree of attention and, therefore, the conceptual use of the activity. In the Creativity and Imagination area, the mean score in Imagination (3.38) is higher than that of Creative Expression (2.95). This result could be primarily explained by the tendency to reproduce peers' expressions when working in a group. In terms of Social Interaction and Independent Use, the children showed a high degree of predisposition to collaborate with each other (3.9). However, when it came to showing individual robot programming skills, some children required the adult's help, so the mean score was lower than the rest of the scores (3.5).

In the final evaluation, each child was again asked to make five associations between a hyperonym and its corresponding hyponym, replicating the initial assessment. Tables 2, 3 and 4 compare the success and failure typology before and after the robotic activity. Statistical analyses were performed using the *t*-test carried out with the SPSS software.

**Table 2:** *t*-results of learner’s pre–posttest on correct answers

	*N*	Mean	*t*-value	DF	Sig. (one-tailed)
Pretest	21	2.8095	8.756	20	< .001**
Posttest	21	3.7619	1.620	20	

**p < .01

**Table 3:** *t*-results of learner’s pre–posttest on related semantic mistakes

	*N*	Mean	*t*-value	DF	Sig. (one-tailed)
Pretest	21	.8571	4.602	20	< .001**
Posttest	21	.3333	2.646	20	

**p < .01

**Table 4:** *t*-results of learner’s pre–posttest on non-related semantic mistakes

	*N*	Mean	*t*-value	DF	Sig. (one-tailed)
Pretest	21	1.2857	5.582	20	< .001**
Posttest	21	.8571	5.403	20	

**p < .01

In 71.42% of cases, students made fewer errors in lexical relations after the educational activity with robotics: They went from 56.19% correct results in the initial activity to 75.24% in the final assessment. In addition, students made fewer unrelated errors regarding the type of errors committed (Tables 3, 4), and the decrease in unrelated errors was significant. When students selected the incorrect concept after the robotics activity, they tended to select the semantically related concept. The qualitative analysis shows that, in the initial evaluation, the errors not semantically related to the hyperonym comprised 17.14% of the incorrect answers, while in the final assessment, they decreased to 6.67%. Errors due to selecting a related answer utilizing a seme have been significant: They have gone from representing 25.71% in the initial assessment to 17.14% in the final assessment. These results suggest that the students have integrated, for the most part, the mechanisms of semantic categorization and lexical relation.

## Conclusion

An applied proposal for integrated learning based on the use of educational robotics has been proposed in which 21 four-year-old students applied twenty-first-century competencies (collaboration, creativity, critical thinking, and communication) to achieve curricular learning corresponding to lexical relations, under the heading of “Communication and Representation of Reality.” The study design took into account the issues identified by Tlili et al. (2020) on choosing an appropriate robot according to the age of the students and the design of activities in which ER could serve as a didactic strategy for the achievement of a goal appropriate to the needs of the students. In this case, we selected the Super.Doc robot and set a didactic objective so that the ER-based activity would help internalize an important curricular content in the process of language acquisition and development.

To assess the conclusions of our study, we answer the questions we posed at the beginning of the research:

1. What knowledge has implemented educational intervention mediated by the use of educational robotics?

The learning results have been significant in all cases. These results suggest that the benefits of using the robotics-based activity are centered on meaningful learning and the designed learning context that facilitates students' understanding of the lexical relationships established between concepts. In the pretest activity, an explanation was given as to how the concepts were related to each other. Students were limited to considering the possible lexical relationships between the selection presented. However, in the ER activity, students practiced different cognitive and communicative skills to solve the challenge posed. This challenge was located in a concrete situational context, which undoubtedly helped students internalize the semantic relationships between concepts. This contextual and related learning explains why students produced fewer response failures in the posttests. Furthermore, when examining the typology of errors, it was found that the number of semantically unrelated responses decreased significantly in the posttest phase, and for the most part, they were become semantically related errors, indicating that students, for the most part, understood how lexical relations are established.

(2) What didactic objectives related to the curriculum can be utilized in a crosscutting manner thanks to educational robotics?

Thanks to ER, all the classroom competencies and curriculum content can be applied in an integrated manner. The design of applied activities enables students to work in groups to solve challenges and, in this way, develop transversal skills such as critical thinking and leadership abilities. In our proposal, we assessed the degree of performance shown by each student in three categories of the so-called twenty-first-century competencies: Thinking and Learning, Creativity and Imagination, and Social Interaction and Independent Use.

The category where students scored the highest was *Thinking and Learning*. The scores obtained by the students in *Inquiry* and *Constructing Knowledge* categories reflect the high degree of interest and motivation engendered by the ER-based activity, which subsequently translated into a high degree of conceptual assimilation. The *Problem-Solving* category obtained the lowest scores in the category, which could be explained by the sexually heterogeneous composition of the working groups. As pointed out in the study by Sun et al. (2022), male students show more applied thinking, and female students show more skills in communication and selection of the most effective solution.

In the *Creativity and Imagination* category, the score in *Imagination* stands out. The group work arrangement enabled students to be creative in devising solutions to challenges. However, this same arrangement determines the score in the *Expressive Communication* category since the linguistically more capable students who take on the role of communicative leaders monopolize the speaking turns of the other group members. Finally, in the *Social Interaction and Independent Use* category, the students showed a high degree of intergroup collaboration. However, when programming the robot, they showed little familiarity with robotics as a regular classroom teaching tool and requested the adult's help to program the robot.

(3) What are the characteristics of young children's learning process?

If we analyze the results of our study, two important factors have been highlighted that should be considered when planning didactic proposals with ER. The first factor refers to the curricular design of the knowledge; specifically, the applicability of the contents to a real context where students can transfer knowledge from conceptual abstraction to its internalization through practical application must be assessed. The second factor is related to the importance of the ER activity in activating the crosscutting cognitive and linguistic skills that are part of the so-called twenty-first century.

There are still some limitations to the implementation of this study. Firstly, the time for carrying out the activity was limited by the school's schedule and calendar. It could have been possible to better assess the students' performance and internalization of the strategies and contents after an intervention with more sessions, but this was not possible. Secondly, although the researchers conducted the ER-based activity, both the initial and the final activities were assessed by teachers as the teachers did not consider themselves sufficiently trained in ER to carry out the activity. In addition, the examiners' access to the educational center was restricted due to COVID-19 restrictive measures. Third, the sample size of our study just allows us to describe a trend in favor of using ER as a powerful resource to achieve learning outcomes. Nevertheless, our study paves the way for future research that, with a larger number of participants, can not only better reflect the effects of our language learning activities, but also establish, in a constructivist frame, a firm link between the pedagogical use of ER and its learning benefits, always oriented toward the development of dynamic and strategic skills working in a problem-based learning environment. Finally, this study has not considered the influence of variables such as gender, level of oral proficiency, or previous level of lexical knowledge. Future research should consider different variables and unify the data collection and evaluation process to be performed by researchers.

## Data Availability

The datasets generated and/or analyzed during the current study are available in the OSF Registries repository Data: osf.io/m6u9e.

## Appendix 1: TEPI’s results

**Table:**

Student ID	Thinking and learning				Creativity and imagination		Social interaction and independent use	
	Constructing knowledge	Problem-solving	Inquiry	Engagement	Creative express	Imagination	Collaboration	Independent use
1	4	5	5	5	4	4	5	5
2	3	3	4	3	3	2	3	2
3	4	4	4	4	3	4	4	4
4	3	3	3	2	3	3	3	2
5	4	3	4	3	3	4	3	3
S	3	2	3	3	2	2	3	2
7	4	3	4	3	2	3	4	4
3	4	4	4	3	4	3	4	4
9	5	5	5	5	5	5	4	5
10	3	4	4	3	3	3	2	3
11	4	3	4	4	3	3	4	3
12	5	4	4	4	3	3	5	4
13	2	1	1	2	1	2	3	1
14	3	4	4	4	2	3	4	4
15	5	5	5	4	3	5	5	5
16	5	4	5	4	3	5	5	5
17	5	5	5	5	4	4	5	5
IS	3	3	4	4	3	2	4	3
19	4	3	4	4	2	4	4	4
20	3	3	4	4	3	4	4	3
21	3	3	3	4	3	3	4	3
Media	3.76	3.52	3.95	3.67	2.95	3.38	3.90	3.52
